# Health Tract, No. 244

**Published:** 1866-06

**Authors:** 


From the Boston Watchman and Reflector, by Dr. W. W. Hall, M. D.
HEALTH TRACT, No. 244.

### CURIOSITIES OF BREATHING.

The taller men are, other things been eqnal, the more lungs they have and the greater number of cubic inches of air they can take in or deliver, at a single breath. It is generally thought that a man's lungs are sound and well developed, in proportion to his girth around the chest, yet observation shows that slim men as a rule will run faster, and farther, with less fatigue having "more wind," than stout men. If two persons are taken, in all respects alike except that one measures twelve inches more around the chest than the other, the one having the excess will not deliver more air at one full breath, by mathematical measurement, than the other.

The more air a man receives into his lungs in ordinary breathing, the more healthy he is likely to be; because an important object in breathing is to remove impurities from the blood. Each breath is drawn pure into the lungs; on its outgoing the next instant it is so impure, so perfectly destitute of nourishment, that if rebreathed without any admixture of a purer atmosphere, the man would die. Hence, one of the conditions necessary to secure a high state of health is, that the rooms in which we sleep should be constantly receiving new supplies of fresh air through open doors, windows or fireplaces.

If a person's lungs are not well developed the health will be imperfect, but the development may be increased several inches in a few months by daily out-door runnings with the mouth closed, beginning with twenty yards and back, at a time, increasing ten yards every week, until a hundred are gone over, thrice a day. A substitute for ladies and persons in cities, is running up stairs with the mouth closed, which compels very deep inspirations, in a natural way, at the end of the journey.

As consumptive people are declining, each week is witness to their inability to deliver as much air at a single out-breathing as the week before, hence the best way to keep the fell disease at bay is to maintain lung development.

It is known that in large towns, ten thousand feet above the level of the sea, the deaths by consumption are ten times less than in places nearly on a level with the sea. Twenty-five persons die of consumption in the city of New York, where only two die of that disease in the city of Mexico. All know that consumption does not greatly prevail in hilly countries and in high situations. One reason of this is because there is more ascending exercise, increasing deep breathing; besides, the air being more rarified, larger quantities are instinctively taken into the lungs to answer the requirements of the system, thus at every breath keeping up a high development. Hence the hill should be sought by consumptives, and not low flat situations.

